# Double jeopardy: implications of neurodevelopmental conditions and adverse childhood experiences for child health

**DOI:** 10.1007/s00787-022-02081-9

**Published:** 2022-09-26

**Authors:** Ruchika Gajwani, Helen Minnis

**Affiliations:** 1 Institute of Health and Wellbeing, Academic CAMHS, West Glasgow Ambulatory Care Hospital, University of Glasgow, Glasgow, G3 8SJ UK; 2 NHS Greater Glasgow and Clyde, Glasgow, UK

## Introduction

### Understanding developmental pathways to child health

Addressing the health needs of people suffering from multiple health conditions, including those with complex psychiatric presentations, is an increasing global challenge [1]. Complex presentations are associated with adverse childhood experiences (ACEs) or trauma, but the focus on trauma and ACEs risks a loss of focus on heritable or temperamental factors that can shape the environment the child grows up in [2]. One such factor, often overlooked, is the presence of neurodevelopment conditions. Recent criticism of the term “neurodevelopmental disorders” [3] has pointed out that we all have neurodevelopment, and what we traditionally refer to as ‘neurodevelopmental disorders’ encompasses traits that are not entirely disadvantageous. For this reason, we use the term ‘neurodevelopmental conditions’ to avoid value judgements, while differentiating conditions such as ADHD and ASD from the neurodiversity that is in all of us.

The potential to overlook neurodevelopmental conditions is particularly high if the child is experiencing adversity [4], and the existence of ACEs can delay the diagnosis of conditions such as Autism Spectrum Disorder (ASD) and Attention-deficit Hyperactivity Disorder (ADHD) [5]. Here we argue that psychiatrists must maintain a focus on *both* ACEs *and* neurodevelopmental conditions, since not doing so can place their patients at double jeopardy of poor health outcomes.

## Adverse childhood experiences increase the risk of poor child health outcomes

ACEs, especially child maltreatment, greatly increase the risk of a wide range of poor health and psychosocial outcomes in a dose–response relationship: the more adversity an individual has experienced in early childhood, the higher the risk of negative health outcomes in adulthood [4, 6]. Yet, many people who have experienced childhood adversity do not go on to have poor health outcomes [4]. Is the case for ACEs *causing* poor health outcomes proven? Although plausible causal pathways have been suggested, recent evidence from longitudinal studies, some of which are genetically sensitive [7, 8], suggest these causal pathways are far from simple.

## Neurodevelopmental conditions increase the risk of poor child health outcomes

Neurodevelopmental conditions (NDCs) such as ASD, ADHD and Intellectual Disabilities are now known to be lifelong conditions, although their manifestations may change across development. NDCs do not necessarily lead to poor outcomes: in the right environment, people with neurodevelopmental conditions can thrive [8]. Yet NDCs are known risk factors for poor mental and physical health, as well as premature mortality. Although the neurodevelopmental roots of severe psychiatric disorders such as schizophrenia and bipolar disorder have long been the focus of intense research scrutiny, the potential role of common neurodevelopmental conditions is often disregarded during mental health assessments in adults.

## ACEs and neurodevelopmental conditions often co-exist and can have additive effects on child health

Child maltreatment and neurodevelopmental conditions often co-exist [9], as we have demonstrated [8]: using a general population twin sample involving several thousand 9-year-olds, we asked whether children exposed to maltreatment have an increased neurodevelopmental condition (NDC) “load” (i.e. symptoms of ADHD, ASD, Tic Disorders and Intellectual Disabilities) compared to children not exposed to maltreatment. We found that maltreated children were nearly ten times as likely as their non-maltreated peers to have symptoms of neurodevelopmental conditions in three or more of the four symptom clusters we investigated—yet maltreatment did not cause this increased neurodevelopmental load [8]. We have also recently shown, in a large general population study, that adolescents are at twice the risk of developing symptoms of severe psychiatric disorder (in this case mania) if, at age 9, they had experience of *both* child maltreatment *and* symptoms of neurodevelopmental conditions(s) [10].

## Could stress physiology be the key mechanism?

We suspect that calibration of stress responses might be a key mechanism underpinning this additive effect. The Adaptive Calibration Model proposes that some degree of adversity in early life is normative, and that organisms make biological adjustments in the face of adversity, even if the stress was very severe as in the case of abuse or neglect [11]. These adaptations can prepare us to cope with further stress in the environment and can have positive effects, for example, the development of “hidden talents” such as exceptional creativity. Yet, if our ability to adapt does become overwhelmed, the major population “killers” can arise later in life e.g., heart disease, cancer, violent behavior and suicide. Child maltreatment and other forms of violent victimization are fundamental human rights violations yet are common experiences: although rates vary widely across the world, the WHO estimates that more than half of all the world’s children have experienced violent victimization. Despite a wide variation in the prevalence of child maltreatment internationally, the rates of severe mental illness are fairly stable across the globe [12], so clearly the causal relationship between abuse and severe mental illness is not a simple one.

During any acutely stressful episode, the autonomic nervous system determines the degree and rate of increase in pulse and blood pressure and there is an outpouring of stress hormones such as adrenalin and cortisol. There are recognizable patterns in the way individuals respond to stress and these patterns have variable associations with psychopathology. The ordinary, intermittent stress that most of us experience throughout life usually results in “moderate” stress responses, which are ideal for coping with ordinary stresses. If, however, we have experienced either severe adversities like maltreatment [13], or hardly any stress at all, we might develop “hyper-responsivity” (a larger, faster increase in pulse, blood pressure and stress hormone secretion) when faced with ordinary life stresses [13]. Some individuals, especially those who have experienced severe or repeated adversity, develop “hypo-responsivity”, with less of an increase in pulse, blood pressure and stress hormones [13]. The ability of human stress response systems to adapt to different environments is undoubtedly crucial for allowing us to survive and thrive in a range of difficult circumstances, but some individuals are more prone than others to their stress response systems becoming overwhelmed. When something stressful happens, the autonomic nervous system prepares the organism for “fight, flight or freeze”, i.e., to attack, escape or become inconspicuous. In turn, this stimulates the production of immune markers e.g. C-Reactive Protein, CRP and Tumor Necrosis Factor alpha, TNF-a, in case there is also a need to fight injury or disease.

Individuals differ in their susceptibility to environmental stressors [14], and the way each individual responds to acute stress may partly depend on temperament, for example, whether a person is naturally fearless or not. Some of the temperamental traits that influence stress responsivity include symptoms of neurodevelopmental conditions, e.g., the impulsivity characteristic of ADHD and the sensory sensitivity common in ASD. The mechanisms underpinning these neurodevelopment-related problems with stress calibration are unknown and might simply be due to the symptom profile that characterizes these disorders. For example, children with ADHD are more likely than their peers to experience stress linked to forgetting homework, missing what the teacher has just said in class or impulsively saying/doing things that get them into trouble—and their impulsivity might also lead to temper tantrums which will, in turn, undoubtedly make their environment even more stressful. In ASD, perfectionism and sensory sensitivities might lead to meltdowns when routines are interrupted or when sensations (e.g., certain noises, textures or tastes) become intolerable. Protective factors might also have an important role here: for example, a child with sensory sensitivities and an intolerance of noise in the classroom might benefit from ear guards, so that their stress calibration remains adaptive and leads to positive development.

The reasons behind the association between maltreatment and neurodevelopmental conditions is unknown and is currently the focus of intense scientific scrutiny: we suspect that adaptive calibration of stress physiology is crucial here. Parents of children with neurodevelopmental conditions are more likely to experience stress or even burn-out, and parental stress is a major risk factor for child maltreatment: the stress systems of family members are linked such that the autonomic reactivity of the infant is closely correlated with the autonomic reactivity of the parents. Although maltreatment does not appear to cause neurodevelopmental conditions, the onset of maltreatment in a child who already has a neurodevelopmental condition is likely to worsen NDC symptoms [8].

The Stress-Diathesis model, in which stressors are thought to interact with inherent vulnerabilities (i.e. diatheses) to produce disease was first posited in the 1950s, and the interaction between predispositions and stressful circumstances has been a fundamental concept in psychiatry for well over a century. Stress-diathesis phenomena have also been described in a wide range of physical illnesses including diabetes, cancers and acute cardiovascular events. The stress side of the stress-diathesis model is well understood: the role of stressful life events such as childhood maltreatment in disease risk, particularly in psychosis research [15] is well recognized. However, the *nature of the diatheses* has never been satisfactorily described: we propose that they are common neurodevelopmental conditions such as ADHD, ASD and Intellectual Disabilities which have been hiding in plain sight all along.

## The double jeopardy model: a reminder to think about both ACEs and NDCs

**Fig. 1:**
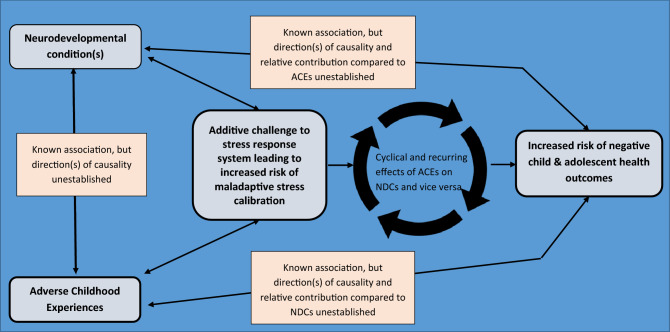
Double jeopardy: a developmental model delineating the role of adverse child experiences (ACEs) and neurodevelopmental disorders (NDDs) for child health

The new understanding emerging from the Adaptive Calibration Model [11] and the complex relationship between ACEs/trauma and NDCs has stimulated us to propose a new model that might provide a useful framework for examining health risk across the lifespan. We have called it Double Jeopardy because it considers the increased health risks associated with *both* adverse childhood experiences *and* neurodevelopmental conditions—and because both ACEs and NDCs are known to increase risk of maladaptive stress calibration (see Fig. 1).

Important areas for further investigation include:

- Timing, i.e. are there specific times across the life course when individuals with neurodevelopmental conditions are more vulnerable to the impact of trauma?
- What protective factors might ameliorate the impact of trauma in those with neurodevelopmental conditions, and are there aspects of neurodevelopmental conditions that might protect against the negative impacts of trauma?

## Implications for psychiatry

Psychiatrists are beginning to fully embrace neurodevelopmental conditions but may have training needs regarding assessment of the full range of neurodevelopmental conditions, including Intellectual Disability, especially when there is a history of ACEs or trauma. If Double Jeopardy is embraced, then there are important implications for service design, including a move away from supra-specialization and a move toward holistic assessment that takes account of both ACEs and NDCs. This would ensure that patients with complex presentations are not misdiagnosed, nor fail to receive the comprehensive assessments that should ensure they receive the best psychiatric care.
